# Perivascular spaces are associated with tau pathophysiology and synaptic dysfunction in early Alzheimer’s *continuum*

**DOI:** 10.1186/s13195-021-00878-5

**Published:** 2021-08-05

**Authors:** Natalia Vilor-Tejedor, Iacopo Ciampa, Grégory Operto, Carles Falcón, Marc Suárez-Calvet, Marta Crous-Bou, Mahnaz Shekari, Eider M. Arenaza-Urquijo, Marta Milà-Alomà, Oriol Grau-Rivera, Carolina Minguillon, Gwendlyn Kollmorgen, Henrik Zetterberg, Kaj Blennow, Roderic Guigo, José Luis Molinuevo, Juan Domingo Gispert, for the ALFA study, Annabella Beteta, Anna Brugulat, Raffaele Cacciaglia, Alba Cañas, Carme Deulofeu, Irene Cumplido, Ruth Dominguez, Maria Emilio, Karine Fauria, Sherezade Fuentes, Laura Hernandez, Gema Huesa, Jordi Huguet, Paula Marne, Tania Menchón, Albina Polo, Sandra Pradas, Blanca Rodriguez-Fernandez, Aleix Sala-Vila, Gonzalo Sánchez-Benavides, Gemma Salvadó, Anna Soteras, Marc Vilanova

**Affiliations:** 1 Barcelonaβeta Brain Research Center (BBRC), Pasqual Maragall Foundation, Barcelona, Spain; 2 Centre for Genomic Regulation (CRG), The Barcelona Institute for Science and Technology, Barcelona, Spain; 3 Department of Clinical Genetics, Erasmus Medical Center, Rotterdam, The Netherlands; 4 Universitat Pompeu Fabra, Barcelona, Spain; 5 Department of Radiology, Hospital Universitari Sagrat Cor, Barcelona, Spain; 6 IMIM (Hospital del Mar Medical Research Institute), Barcelona, Spain; 7 Centro de Investigación Biomédica en Red de Fragilidad Y Envejecimiento Saludable (CIBERFES), Madrid, Spain; 8 Centro de Investigación Biomédica en Red Bioingeniería, Biomateriales Y Nanomedicina, Madrid, Spain; 9 Servei de Neurologia, Hospital del Mar, Barcelona, Spain; 10 Department of Epidemiology, Harvard T.H. Chan School of Public Health, Boston, MA USA; 11 Cancer Epidemiology Research Program, Catalan Institute of Oncology (ICO), Hospitalet del Llobregat, Spain; 12 Roche Diagnostics GmbH, Penzberg, Germany; 13 Department of Psychiatry and Neurochemistry, Institute of Neuroscience and Physiology, University of Gothenburg, Mölndal, Sweden; 14 Clinical Neurochemistry Laboratory, Sahlgrenska University Hospital, Mölndal, Sweden; 15 Department of Neurodegenerative Disease, UCL Institute of Neurology, Queen Square, London, UK; 16 Dementia Research Institute At UCL, London, UK

**Keywords:** Alzheimer’s disease, MRI, CSF biomarkers, Perivascular spaces, Tau pathophysiology, Virchow-Robin spaces

## Abstract

**Background:**

Perivascular spaces (PVS) have an important role in the elimination of metabolic waste from the brain. It has been hypothesized that the enlargement of PVS (ePVS) could be affected by pathophysiological mechanisms involved in Alzheimer’s disease (AD), such as abnormal levels of CSF biomarkers. However, the relationship between ePVS and these pathophysiological mechanisms remains unknown.

**Objective:**

We aimed to investigate the association between ePVS and CSF biomarkers of several pathophysiological mechanisms for AD. We hypothesized that ePVS will be associated to CSF biomarkers early in the AD continuum (i.e., amyloid positive cognitively unimpaired individuals). Besides, we explored associations between ePVS and demographic and cardiovascular risk factors.

**Methods:**

The study included 322 middle-aged cognitively unimpaired participants from the ALFA + study, many within the Alzheimer’s *continuum*. NeuroToolKit and Elecsys® immunoassays were used to measure CSF Aβ42, Aβ40, p-tau and t-tau, NfL, neurogranin, TREM2, YKL40, GFAP, IL6, S100, and α-synuclein. PVS in the basal ganglia (BG) and centrum semiovale (CS) were assessed based on a validated 4-point visual rating scale. Odds ratios were calculated for associations of cardiovascular and AD risk factors with ePVS using logistic and multinomial models adjusted for relevant confounders. Models were stratified by Aβ status (positivity defined as Aβ42/40 < 0.071).

**Results:**

The degree of PVS significantly increased with age in both, BG and CS regions independently of cardiovascular risk factors. Higher levels of p-tau, t-tau, and neurogranin were significantly associated with ePVS in the CS of Aβ positive individuals, after accounting for relevant confounders. No associations were detected in the BG neither in Aβ negative participants.

**Conclusions:**

Our results support that ePVS in the CS are specifically associated with tau pathophysiology, neurodegeneration, and synaptic dysfunction in asymptomatic stages of the Alzheimer’s *continuum*.

**Supplementary Information:**

The online version contains supplementary material available at 10.1186/s13195-021-00878-5.

## Background

Perivascular spaces (PVS) [1, 2], also known as Virchow-Robin spaces, facilitate CSF transport from the basal cisterns into the brain interstitial fluid (ISF) [3] and have an important role in the elimination of metabolic waste and fluid from the brain [4]. Interestingly, water influx into the CSF flow through PVS could play a role equivalent to the one in the lymphatic system and it recently gained substantial attention due to its relation to amyloid-β (Aβ) clearance [5–7]. Indeed, enlargement of PVS (ePVS) may result in impaired ISF drainage and has been shown to be associated with cerebral amyloid angiopathy in cognitively impaired patients [8, 9], and with peripheral neuroinflammatory biomarkers in the elderly [10, 11]. The impairment of this biological mechanism might contribute to the development of AD pathophysiology, characterized by Aβ plaques and tau tangles. On top of these, several other pathophysiological mechanisms have been shown to be altered in preclinical AD stages [12].

The main hypothesis of our study was to investigate whether altered levels of CSF AD biomarkers were associated with ePVS in a sample of middle-aged cognitively unimpaired participants, many within the Alzheimer’s *continuum.* The term “Alzheimer’s continuum” is applied as an umbrella term that includes both “Alzheimer’s pathological change” and “Alzheimer’s disease” as defined in the NIA-AA Research Framework nomenclature [13]. The term “Alzheimer’s pathologic change” is used when there is evidence of Aβ pathology but not tau, whereas the term “Alzheimer’s disease” is applied whenever there is evidence of both Aβ and tau pathology, regardless of the clinical manifestations.

We first explored the association between PVS with demographic and cardiovascular-related risk factors and assessed the dependency of the results with Aβ status. Moreover, we sought for associations between PVS with additional CSF biomarkers of several pathophysiological mechanisms involved in AD and other neurodegenerative disorders such as axonal damage (NfL), synaptic dysfunction (neurogranin), microglial (sTREM2), astroglial-related response (GFAP, YKL40, S100), other neuroinflammatory biomarkers (IL6), and α-synuclein.

## Material and methods

### Participants

**Fig. 1:**
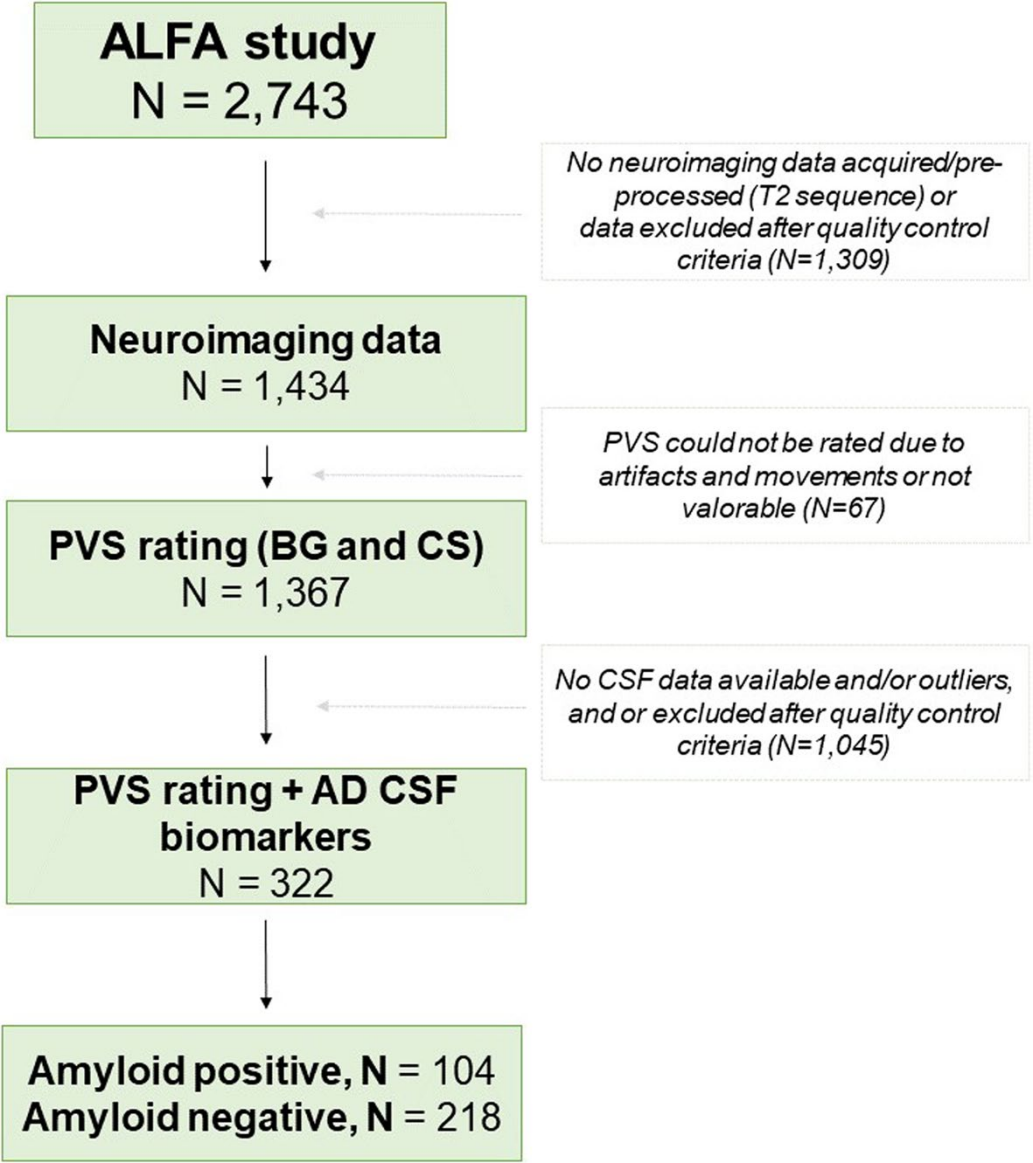
Flow chart that shows the selection of the participants of the study. ALFA study, ALzheimer and FAmilies; PVS, perivascular spaces; CSF, cerebrospinal fluid; BG, basal ganglia; CS, centrum semiovale; N, sample

The study included participants from the ALFA study (Alzheimer and Families) at the Barcelonaβeta Brain Research Center [14], which aims at studying the preclinical stage of AD. The ALFA study (Clinicaltrials.gov Identifier: NCT01835717) includes 2743 cognitively unimpaired participants, including a high proportion of AD patients’ offspring, aged between 45 and 75 years. In this study, a subset of 322 participants from a nested study (ALFA + ; NCT02485730) was included. ALFA + individuals were invited based on their specific AD risk profile. This AD profile was determined by an algorithm in which participants’ AD parental history, age, number of *APOE*-ε4, alleles, verbal episodic memory score, and CAIDE (Cardiovascular Risk Factors, Aging, and Incidence of Dementia) score were taken into consideration. These individuals are cognitively unimpaired and, therefore, are in the preclinical stage of the Alzheimer’s continuum. In terms of main demographic characteristics, the percentages do not differ from the ALFA parent cohort**.** In addition, all individuals included in this study have available information on *APOLIPOPROTEIN E (APOE)* genotype, MRI examination, CSF biomarker levels, as well as cardiovascular risk factors (Fig. 1). Notice that the average time range between MRI acquisition and CSF sampling was 44 days (± 57 days). MRI acquisition and cognitive assessment were performed at the same visit.

### Image acquisition

Scans were obtained with a 3 T scanner (Ingenia CX, Philips, Amsterdam, Netherlands). The MRI protocol was identical for all participants and included high-resolution 3D T2-weighted image: Turbo Spin Echo (TSE), voxel size 1 × 1x1 mm^3^, repetition time/echo time (TR/TE): 2500/264 ms, flip angle = 90°. In addition, a 3D T1-weighted Turbo Field Echo (TFE) sequence was acquired (voxel size 0.75 × 0.75 × 0.75 mm, TR/TE: 9.90/4.6 ms, flip angle = 8°) as well as a 3D T2-FLAIR sequence (TSE, voxel size 1 × 1x1 mm, TR/TE/TI: 5000/312/1700 ms). Scans were visually assessed for quality and incidental findings by a trained neuroradiologist. T1w images were segmented to compute total gray matter volume and total intracranial volume (TIV) using Freesurfer 6.0 (https://surfer.nmr.mgh.harvard.edu/). In addition, white matter hyperintensities (WMH) were segmented from FLAIR images using the Lesion Segmentation Toolbox (LST; https://www.applied-statistics.de/lst.html) for SPM12 [15].

### Rating of perivascular spaces

**Fig. 2:**
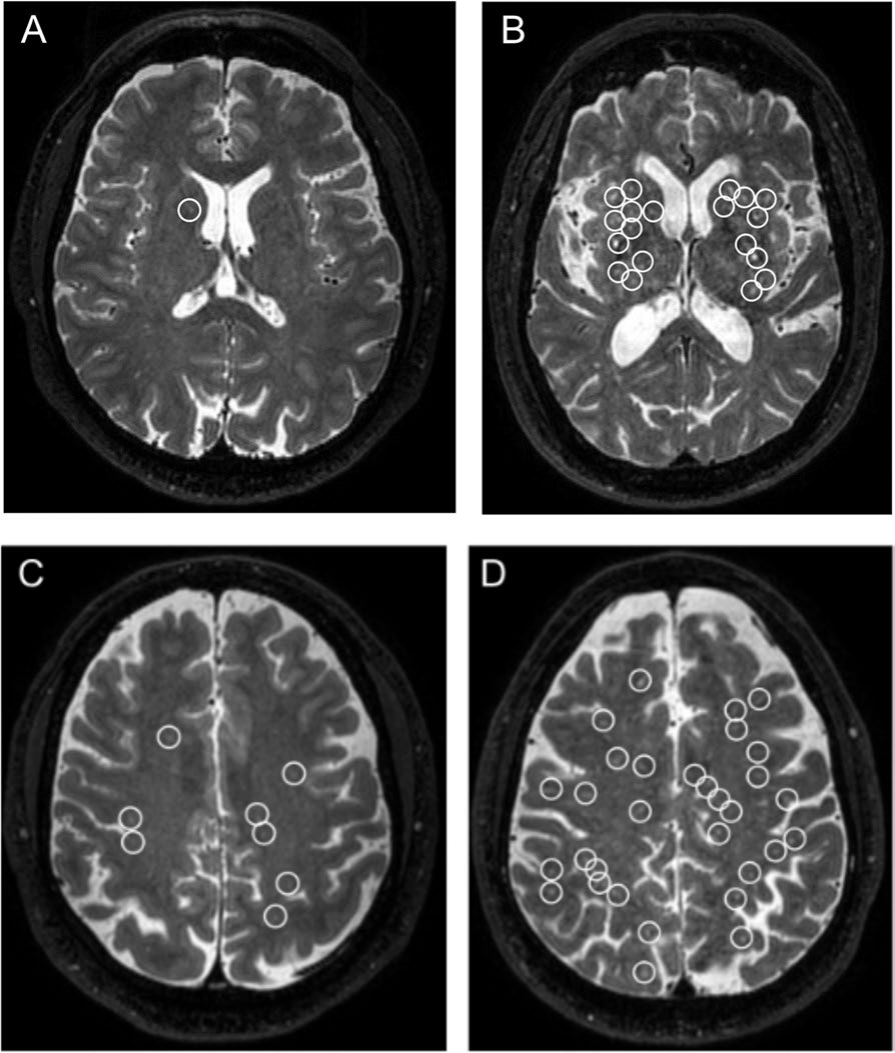
Perivascular spaces rating. **A** Score of 1 in basal ganglia (non-severe). **B** Score of 4 in basal ganglia (severe). **C** Score of 1 (non-severe) in centrum semiovale. **D** Score of 4 (severe) in centrum semiovale. Circle: an enlarged Perivascular Space

PVS were evaluated by a radiologist using the visual rating scale developed by [16] based on T2-weighted images. The radiologist was blind to clinical assessment and quantification of variables of interest used in the study. Briefly, PVSs were quantified independently in two brain regions, including BG, and centrum semiovale (CS). PVS in BG and CS were assessed in the slice and hemisphere with the highest number and rated as 0 (no PVS; degree 0), 1 (mild; 1–10 PVS; degree 1), 2 (moderate; 11–20 PVS; degree 2), 3 (frequent; 21–40 PVS; degree 3), or 4 (severe; > 40 PVS; degree 4). Examples of the PVS rating are given in Fig. 2. Participants were dichotomized according to the severity of the ePVS rating of the BG and CS (degrees 0–2 were categorized as non-severe or 0, degrees 3–4 were categorized as severe or 1).

The intra-rater agreement rate (Cohen’s Kappa) of PVS rating was evaluated by estimating the intraclass correlation of two independent ratings from the same radiologist on a random sample of 20% of the subjects in the dataset. The intra-rater agreement analysis showed substantial reliability (*κ* = 0.77, *p* = 6.02e − 08 for BG subscale; and *κ* = 0.76, *p* = 8.2e − 10 for CS subscale).

### CSF collection and measurement

The collection of CSF and measurement of biomarkers in ALFA + was previously described comprehensively [12]. In brief, CSF t-tau and p-tau were measured using the electrochemiluminescence immunoassays Elecsys® Total-tau CSF and phosphor-tau(181P) CSF on a fully automated cobas e601 instrument (Roche Diagnostics International Ltd.). The rest of the CSF biomarkers were measured with robust prototype assay as part of the NeuroToolKit (Roche Diagnostics International Ltd, Rotkreuz, Switzerland) on both cobas e 601 and e 411 instruments. All measurements were performed at the Clinical Neurochemistry Laboratory, Sahlgrenska University Hospital, Mölndal, Sweden.

Aβ pathology positivity (Aβ +) was defined by CSF Aβ42/40 ratio. We derived the cutoffs for each of these biomarkers using a two-Gaussian mixture modeling. The cut-off was defined as the mean plus 2 standard deviations (SD) of the non-pathologic Gaussian distribution (i.e., the Gaussian with the higher mean value for Aβ42/40 ratio and the resulting cutoff was 0.071. This approach for the definition of Aβ + has been shown to be optimal for the detection of pathophysiological changes in early stages of the Alzheimer’s *continuum* [17]*.* A total of 122 individuals in the study were categorized as Aβ + .

### Risk factors assessment

Sociodemographic and clinical data were collected during face-to-face interviews by trained neuropsychologists, study nurses, and clinical neurologists. Participants' systolic and diastolic blood pressure were measured twice and the second measure was used. Total cholesterol level was obtained from a blood test (lipoprotein panel). Body mass index (BMI) was derived from the height and weight measured at the time of the interview. Physical activity was measured using the Spanish short version of the Minnesota Leisure Time Physical Activity Questionnaire [18] and participants were split into two categories: “active” (more than 150 min of moderate exercise or 75 min of vigorous exercise per week as recommended by the American Heart Association) or ‘inactive’. Participants with systolic blood pressure levels above 140 mmHg, self-reported hypertension diagnosis, or current use of anti-hypertensive medication were considered hypertensive. Diabetes and dyslipidemia status were defined from participant self-reported diagnosis. Moreover, based on these cardiovascular factors, we calculated a dementia risk score CAIDE (Cardiovascular Risk Factors, Aging, and Incidence of Dementia) to be included in the analysis. Further details of the calculation of CAIDE can be found in [19]. The *APOE* allelic variants ε2, ε3, and ε4 were determined from allelic combinations of the rs429358 and rs7412 polymorphisms, where the ε4 allele is the combination of the C allele at both sites [20]. Individuals were classified according to the number of ε4 alleles (non-carriers, heterozygotes, homozygotes). Allele frequencies and departures from Hardy–Weinberg equilibrium were inspected.

### Statistical analysis

Differences between the degree of PVS in demographic and cardiovascular variables were assessed using *χ*^2^ tests (categorical variables), one-way ANOVA (normal continuous variables), and/or non-parametric Kruskal–Wallis tests (non-normal continuous variables).

There were differences in demographics and cardiovascular factors between the degrees of PVS. Therefore, odd ratios were calculated for associations of potential risk factors with ePVS. Associations between CSF biomarkers and ePVS (higher degree of PVS) were examined in logistic regressions (BG-ePVS) and multinomial regressions (CS-ePVS) adjusting for the previous potential confounders selected through stepwise regression (backward method) to generate minimally adjusted models. All regression models were adjusted by age, sex, *APOE*-ɛ4 status, physical inactivity, total GM volume, and TIV. BG-ePVS models were additionally adjusted by systolic blood pressure, and CS-ePVS models were additionally adjusted by diastolic blood pressure.

Analyses with CSF biomarkers were stratified by Aβ status, as defined by CSF Aβ42/40 ratio, to assess whether the relationship between ePVS and CSF biomarker levels differed between individuals with normal (negative) and pathologic (positive) Aβ levels. We first examined associations between ePVS and a non-pathological biomarker (Aβ40) to assess whether ePVS might be associated with overall protein clearance by CSF. We then sought associations between ePVS and the rest of CSF biomarkers with and without correction for Aβ40. This has been the final model selected in the study. All these models were corrected by relevant demographic and cardiovascular factors previously identified.

Notice that, categories of ePVS with less than 20 observations were included in the former category [individuals with degree 3 in BG-ePVS (*N* = 19), and degree 4 in CS-ePVS (*N* = 10). As a post hoc analysis, we excluded these categories, and we reproduced regression models and compared them with previous analyses to evaluate the risk of overfitting (these models produced similar results and were not presented).

Statistical significance was set at *P* ≤ 0.05 and corrected using pair-wise correction (group comparisons) and false discovery rate (FDR) (association models). All statistical analyses and data visualizations were carried out using *R* version 3.6.1.

## Results

### Descriptive

**Fig. 3:**
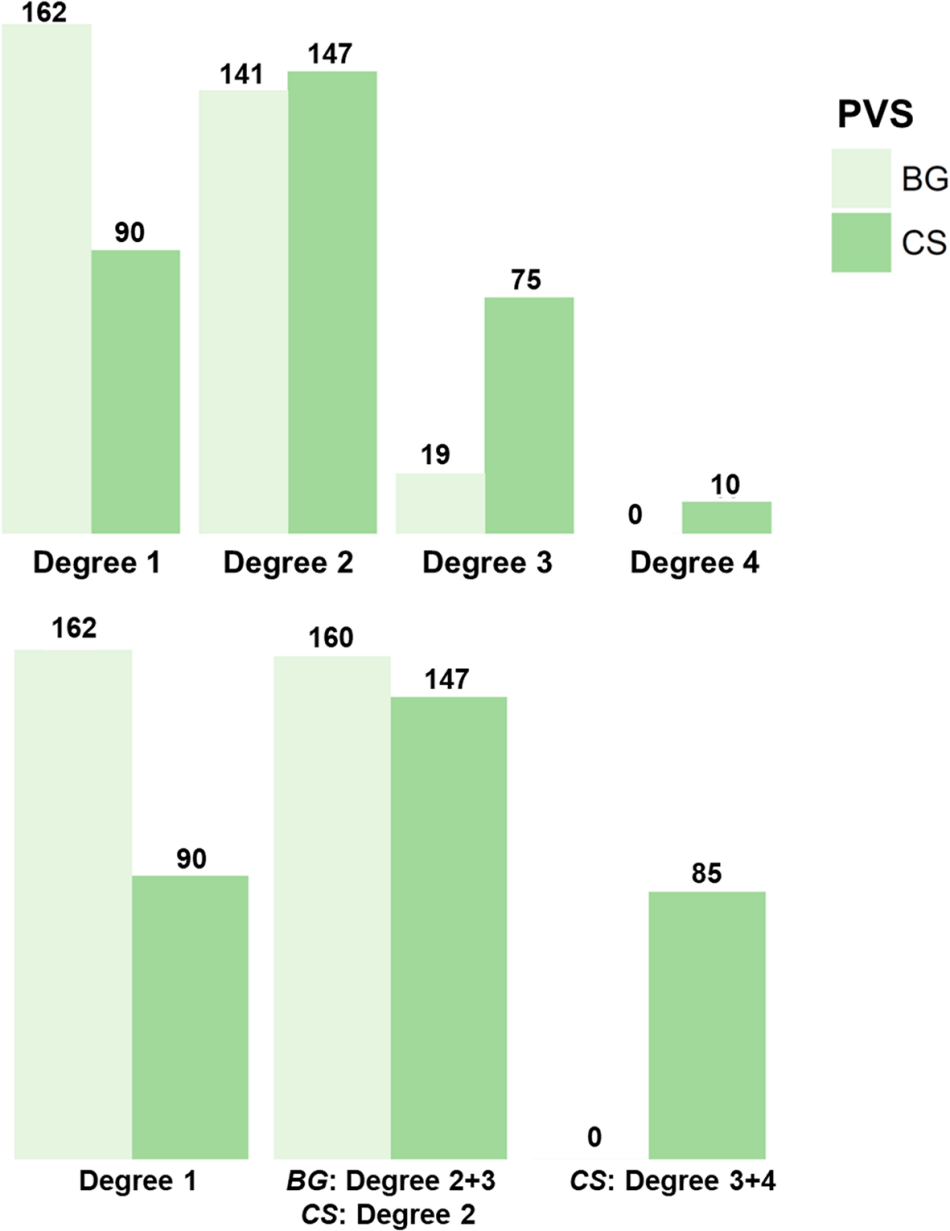
Distribution of Perivascular Spaces by degree of severity. Legend: BG, Basal Ganglia; CS, centrum semiovale. Model 1 shows associations correcting by age, sex and cardiovascular risk factors. Model 2 shows associations correcting by age, sex, cardiovascular risk factors and Aβ40. Blue color intensities indicate the magnitude of the associations (higher levels of biomarkers). Abbreviations: AD, Alzheimer’s disease; PVS, perivascular spaces; BG, basal ganglia; CS, centrum semiovale; CSF, cerebrospinal fluid; Aβ40, amyloid-β 40; Aβ42, amyloid-β 42; GFAP, glial fibrillary acidic protein; IL6, interleukin-6; NfL, neurofilament light; p-tau, phosphorylated tau; sTREM2, soluble triggering receptor expressed on myeloid cells 2 (TREM2); t-tau, total tau

The mean age of the study’s participants was 60.4 (± 4.9) years old, and 64.5% were female. Degree 2 of ePVS was the most frequent in both BG and CS (between 42 and 44% of participants). In contrast, degree 3 in BG (5.7%), and degree 4 in CS (3.01%) were less commonly encountered among participants. In addition, we did not find participants with degree 4 in BG (Fig. 3). Further characteristics of the sample and the distribution of potential risk factors according to ePVS degree are shown in Table 1. Characteristics of the CSF biomarker sample according to ePVS degree are shown in Table 2, and characteristics of the sample stratified by Aβ42/40 status are shown in Supplemental Table 1.

**Table. 1:** Characteristics of the sample and group comparisons of potential risk factors across degrees of Perivascular Spaces in Basal Ganglia and Centrum Semiovale Regions. Legend: *N*, sample size; *n*, count of individuals for each categorical variable; *SD*, standard deviation; *ePVS*, enlarged perivascular spaces; *BG*, basal ganglia; *CS*, centrum semiovale; *CAIDE*, Cardiovascular Risk Factors, Aging and Incidence of Dementia; *BMI*, body mass index; *WMH*, white matter hyperintensities; *GM*, gray matter volume; *TIV*, total intracranial volume

			**BG-ePVS**			**CS-ePVS**			
	***N***	**Mean (SD) or** ***n* (%)**	**Degree 1 (*n* = 162)**	**Degree 2 + Degree 3 (*n* = 160)**	***p* value**	**Degree 1 (*n* = 90)**	**Degree 2 (*n* = 147)**	**Degree 3 + 4 (*n* = 85)**	***p* value**
Sex, *n* (%)	322				0.156				0.549
Male		118 (36.6%)	66 (40.7%)	52 (32.5%)		35 (38.9%)	56 (38.1%)	27 (31.8%)	
Female		204 (63.4%)	96 (59.3%)	108 (67.5%)		55 (61.1%)	91 (61.9%)	58 (68.2%)	
Age (years)	322	60.7 (4.69)	59.4 (4.67)	62.0 (4.37)	** < 0.001**	59.8 (4.76)	60.5 (4.62)	62.0 (4.50)	**0.006**
APOE-ε4 allele status, *n* (%)	322				0.182				0.643
Non-carriers		156 (48.4%)	72 (44.4%)	84 (52.5%)		43 (47.8%)	75 (51.0%)	38 (44.7%)	
Carriers		166 (51.6%)	90 (55.6%)	76 (47.5%)		47 (52.2%)	72 (49.0%)	47 (55.3%)	
Education (years)	318	13.4 (3.50)	13.6 (3.39)	13.2 (3.61)	0.346	13.3 (3.22)	13.8 (3.50)	12.9 (3.76)	0.188
Diabetes, *n* (%)	304				0.531				0.161
No		294 (96.7%)	141 (95.9%)	153 (97.5%)		77 (93.9%)	137 (98.6%)	80 (96.4%)	
Yes		10 (3.29%)	6 (4.08%)	4 (2.55%)		5 (6.10%)	2 (1.44%)	3 (3.61%)	
Dyslipidemia, *n* (%)	304				0.406				0.551
No		159 (52.3%)	81 (55.1%)	78 (49.7%)		47 (57.3%)	71 (51.1%)	41 (49.4%)	
Yes		145 (47.7%)	66 (44.9%)	79 (50.3%)		35 (42.7%)	68 (48.9%)	42 (50.6%)	
BMI (kg/m^2^)	322	27.0 (4.16)	27.5 (4.34)	26.4 (3.91)	**0.014**	27.8 (4.64)	26.8 (3.72)	26.5 (4.27)	0.077
Systolic Blood Pressure (mmHg)	321	133 (15.9)	131 (14.9)	135 (16.4)	**0.007**	131 (14.9)	133 (15.2)	135 (17.7)	0.228
Diastolic Blood Pressure(mmHg)	321	75.4 (9.89)	75.7 (9.56)	75.1 (10.2)	0.541	74.1 (9.80)	75.1 (9.80)	77.2 (9.97)	0.100
Cholesterol (mg/dL)	292	204 (30.8)	204 (29.1)	204 (32.4)	0.871	205 (32.3)	203 (32.1)	203 (27.2)	0.886
Physical Activity, *n* (%)	288				0.089				0.326
Non-active		55 (19.1%)	20 (14.6%)	35 (23.2%)		12 (15.8%)	24 (17.8%)	19 (24.7%)	
Active		233 (80.9%)	117 (85.4%)	116 (76.8%)		64 (84.2%)	111 (82.2%)	58 (75.3%)	
CAIDE score (0–15)	286	6.00 [4.00;7.00]	5.00 [4.00;7.00]	6.00 [4.00;7.00]	0.129	5.00 [4.00;7.00]	5.00 [4.00;6.00]	6.00 [4.75;7.00]	0.089
Total GM (mm^3^)	278	588,244 (49,789)	597,109 (47,771)	580,229 (50,376)	**0.004**	584,964 (48,140)	590,616 (50,255)	587,617 (51,210)	0.726
WMH (ml)	257	3.33 (16.6)	1.43 (1.72)	5.08 (22.9)	0.068	1.39 (1.39)	4.83 (23.7)	2.48 (2.84)	0.346
TIV (mm^3^)	278	1,438,565 (171,034)	1,456,206 (166,776)	1,422,616 (173,823)	0.101	1,427,412 (163,677)	1,442,693 (179,156)	1,443,534 (165,757)	0.792

**Table. 2:** Characteristics of the sample of CSF biomarkers across degrees of perivascular spaces. Legend: *N*, sample size; *n*, count of individuals for each categorical variable; *SD*, standard deviation; *BG*, basal ganglia; *CS*, centrum semiovale; *ePVS*, enlarged perivascular spaces

			**BG-ePVS**			**CS-ePVS**			
	***N***	**Mean (SD)**	**Degree 1 (*n* = 162)**	**Degree 2 + Degree 3 (*n* = 160)**	***p* value**	**Degree 1 (*n* = 90)**	**Degree 2 (*n* = 147)**	**Degree 3 + 4 (*n* = 85)**	***p* value**
Aβ42 (pg/mL)	322	1374 (604)	1287 (520)	1462 (669)	0.009	1321 (637)	1392 (538)	1399 (677)	0.616
Aβ40	322	17.9 (4.94)	16.8 (4.32)	19.0 (5.28)	** < 0.001**	16.5 (4.43)	17.9 (4.55)	19.3 (5.71)	**0.001**
Aβ42/40 cat, *n* (%)	322				0.102				0.121
Negative		200 (62.1%)	93 (57.4%)	107 (66.9%)		50 (55.6%)	100 (68.0%)	50 (58.8%)	
Positive		122 (37.9%)	69 (42.6%)	53 (33.1%)		40 (44.4%)	47 (32.0%)	35 (41.2%)	
p-Tau (pg/mL)	312	16.8 (7.67)	15.4 (6.08)	18.2 (8.77)	**0.001**	15.1 (5.70)	16.4 (6.60)	19.1 (10.2)	**0.002**
t-Tau (pg/mL)	322	202 (73.6)	186 (61.6)	217 (81.4)	** < 0.001**	184 (61.5)	198 (66.1)	227 (90.1)	** < 0.001**
GFAP (pg/mL)	322	7.66 (2.72)	7.46 (3.00)	7.87 (2.39)	0.167	7.22 (2.13)	7.43 (2.55)	8.54 (3.32)	**0.002**
YKL40 (pg/mL)	322	150 (55.3)	136 (45.7)	165 (60.3)	** < 0.001**	139 (52.6)	145 (46.1)	171 (66.7)	** < 0.001**
TREM2 (pg/mL)	322	8.04 (2.28)	7.55 (2.02)	8.54 (2.41)	** < 0.001**	7.73 (2.22)	7.85 (2.18)	8.69 (2.38)	**0.008**
IL6 (pg/mL)	322	4.08 (2.04)	4.20 (2.16)	3.97 (1.90)	0.311	4.12 (1.92)	4.08 (2.35)	4.03 (1.55)	0.960
NFL (pg/mL)	322	83.4 (36.7)	78.8 (27.7)	88.1 (43.6)	0.024	82.5 (48.4)	79.8 (25.5)	90.6 (38.2)	0.093
Neurogranin (pg/mL)	322	816 (339)	740 (299)	893 (359)	** < 0.001**	716 (301)	809 (306)	933 (394)	** < 0.001**
S100 (pg/mL)	322	1.02 (0.24)	1.02 (0.25)	1.03 (0.23)	0.722	0.99 (0.22)	1.01 (0.21)	1.07 (0.29)	0.080
α-Synuclein (pg/mL)	322	245 (277)	212 (180)	278 (347)	0.035	199 (147)	249 (265)	286 (381)	0.108

### Association between demographics and cardiovascular risk factors on ePVS

Table 3 shows associations between demographic and cardiovascular risk factors with BG-ePVS and CS-ePVS. We observed that the degree of PVS significantly increased with age in both, BG and CS regions (BG, OR [CI95%]: 1.14 [1.07, 1.23]; CS, degree 2 vs degree 1, OR [CI95%]: 1.11 [1.01,1.2]; degree 3 + 4 vs 1, OR [CI95%]: 1.27 [1.13,1.45]; degree 3 + 4 vs 2, OR [CI95%]: 1.16 [1.05, 1.28]).

**Table. 3:** Associations between enlargement of perivascular spaces in basal ganglia and centrum semiovale regions, and demographic and cardiovascular risk factors. Legend: *n*, count of individuals for each categorical variable; *SD*, standard deviation; *BG*, basal ganglia; *CS*, centrum semiovale; *ePVS*, enlarged perivascular spaces; *WMH*, white matter hyperintensities; *GM*, gray matter volume; *TIV*, total intracranial volume; *OR*, odds ratio; *CI*, confidence interval

	**BG-ePVS**	**CS-ePVS**		
	**OR (95% CI)**	**OR (95% CI)**	**OR (95% CI)**	**OR (95% CI)**
	**Degree 2 + 3 vs Degree 1**	**Degree 2 vs Degree 1**	**Degree 3 + 4 vs Degree 1**	**Degree 3 + 4 vs Degree 2**
Sex, *n* (%)				
Male	Ref	Ref	Ref	Ref
Female	1.29 [ 0.65, 3.04]	2.32 [0.88, 6.33]	**5.5 [1.59, 21.06]**	3.61 [1.39, 9.92]
Age (years)	**1.14 [1.07, 1.23]**	**1.1 [1.01, 1.20]**	**1.27 [1.13, 1.45]**	**1.16 [1.05, 1.28]**
APOE-ε4 allele carriers, n(%)				
Non-carriers	Ref	Ref	Ref	Ref
Heterozygous	1.07 [0.59, 2.19]	1.03 [0.49, 2.23]	0.86 [0.31, 2.41]	0.99 [ 0.45, 2.11]
Homozygous	1.33 [0.50, 6.34]	0.57 [0.17, 2.01]	0.72 [0.10, 4.03]	5.03 [0.86, 31.43]
Systolic blood pressure (mmHg)	1.02 [0.99, 1.01]	1.02 [0.98, 1.06]		
Diastolic blood pressure (mmHg)			1.06 [1.004, 1.12]	1.02 [0.98, 1.06]
Physical activity, *n* (%)				
Non-active	Ref	Ref	Ref	Ref
Active	0.52 [0.26, 1.27]	0.78 [ 0.31, 1.87]	0.48 [0.13, 1.69]	0.98 [0.41, 2.46]
Total GM (mm^3^)	1.0001 [0.99, 1.001]	1.0001 [0.99, 1.001]	1.0001 [1.0001, 1.001]	1.0001 [0.99, 1.00001]
WMH (ml)	1.17 [1.0001, 1.45]	1.07 [0.91, 1.31]	1.08 [0.82, 1.47]	1.001 [0.99,1.01]
TIV (mm^3^)	1.0001 [0.99, 1.001]	0.999 [0.99, 1.001]	1.0001 [0.99, 1.00001]	1.0001 [0.99, 1.00001]

In the CS region, specific significant predictors of increasing PVS degree were higher diastolic blood pressure, and higher WMH (degree 3 + 4 vs degree 1, OR[CI95%]: 1.02[1.01, 1.03]; degree 3 + 4 vs degree 1, OR[CI95%]: 1.33[1.06, 1.67]) (Supplemental Table 2). However, they were not significant after adjusting for additional risk factors (Table 3). Interestingly, females, who did not show significant associations in individual models, presented higher risk for CS-ePVS after adjusting for cardiovascular risk factors (degree 3 + 4 vs degree 1, OR [CI95%]: 5.5 [1.59, 21.06]). Finally, we did not find a significant association between the risk of ePVS and the number of *APOE*-ε4 alleles.

### Association between CSF biomarkers on ePVS

Models that were not corrected by Aβ40 levels showed that higher levels of p-tau (OR [CI95%]: 1.08 [1.02, 1.15]), t-tau (OR [CI95%]: 1.01 [1.004, 1.01]), YKL40 (OR [CI95%]: 1.02 [1.01, 1.02]), sTREM2 (OR [CI95%]: 1.23 [1.07, 1.43]), and neurogranin (OR [CI95%]: 1.003 [1.001, 1.004]) were significantly associated with ePVS in the BG region. Only higher levels of CSF NfL (OR [CI95%]: 1.04 [1.008, 1.08]) and neurogranin (OR [CI95%]: 1.003 [1.001, 1.005]) were significantly associated with BG-ePVS in Aβ + individuals. In the CS region, we showed that higher levels of p-tau (OR [CI95%]: 1.08 [1.012, 1.16]), t-tau (OR [CI95%]: 1.01 [1.002, 1.018]), and neurogranin (OR [CI95%]: 1.002 [1.001, 1.004]) were significantly associated with ePVS (Supplemental Table 3, Supplemental Figure 1). Moreover, we found significant associations between higher levels of CSF Aβ40 and a higher degree of PVS in both the BG and CS in the whole sample that persisted when stratifying the analyses by Aβ status. After correcting for levels of CSF Aβ40, significant associations between higher levels of CSF p-tau (OR [CI95%]: 1.16 [1.03, 1.35]), t-tau (OR [CI95%]: 1.02 [1.01, 1.04]), and neurogranin (OR [CI95%]: 1.004 [1.001, 1.008]) (degree 2 vs degree 3 + 4) were found in the Aβ + group, only in the CS region (Table 4).

**Table. 4:** Associations Perivascular Spaces in Basal Ganglia and Centrum Semiovale and CSF biomarkers (logistic and multinomial regressions). Models were adjusted by potential demographic and cardiovascular risk factors, as well as, levels of Aβ40. Models were stratified by Aβ42/40 positive status. *Legend: n, sample size; SD, standard deviation; ePVS, enlarged Perivascular Spaces; BG, Basal Ganglia; CS, Centrum Semiovale; NTK, NeuroToolKit; CSF, cerebrospinal fluid.*

	All Individuals			Aβ42/40 positivity (N=122)			Aβ42/40 negativity (N=200)		
*Outcome*	*Biomarker*	*OR*	*IC*	*Biomarker*	*OR*	*IC*	*Biomarker*	*OR*	*IC*
*BG (degree 2+3 vs degree 1)*	*Aβ42/40*	0.059	[0.025, 0.626]	*Aβ42/40*			*Aβ42/40*		
*BG (degree 2+3 vs degree 1)*	*p-Tau (pg/mL)*	0.980	[0.925,1.055]	*p-Tau (pg/mL)*	1.022	[0.937,1.161]	*p-Tau (pg/mL)*	0.887	[0.74,1.061]
*BG (degree 2+3 vs degree 1)*	*t-Tau (pg/mL)*	0.999	[0.991,1.008]	*t-Tau (pg/mL)*	1.004	[0.992,1.022]	*t-Tau (pg/mL)*	0.992	[0.974,1.011]
*BG (degree 2+3 vs degree 1)*	*GFAP (pg/mL)*	0.883	[0.762,0.998]	*GFAP (pg/mL)*	0.873	[0.589,1.254]	*GFAP (pg/mL)*	0.895	[0.758,1.021]
*BG (degree 2+3 vs degree 1)*	*YKL40 (pg/mL)*	1.008	[0.999,1.018]	*YKL40 (pg/mL)*	1.006	[0.987,1.026]	*YKL40 (pg/mL)*	1.010	[0.999,1.023]
*BG (degree 2+3 vs degree 1)*	*TREM2 (pg/mL)*	1.082	[0.917,1.285]	*TREM2 (pg/mL)*	1.127	[0.814,1.583]	*TREM2 (pg/mL)*	1.072	[0.873,1.325]
*BG (degree 2+3 vs degree 1)*	*IL6 (pg/mL)*	1.108	[0.943,1.324]	*IL6 (pg/mL)*	1.192	[0.854,1.708]	*IL6 (pg/mL)*	1.119	[0.918,1.413]
*BG (degree 2+3 vs degree 1)*	*NFL (pg/mL)*	1.000	[0.986,1.014]	*NFL (pg/mL)*	1.031	[0.998,1.07]	*NFL (pg/mL)*	0.991	[0.974,1.009]
*BG (degree 2+3 vs degree 1)*	*Neurogranin (pg/mL)*	1.001	[0.999,1.004]	*Neurogranin (pg/mL)*	1.002	[0.999,1.007]	*Neurogranin (pg/mL)*	1.001	[0.998,1.005]
*BG (degree 2+3 vs degree 1)*	*S100 (pg/mL)*	0.850	[0.177,4.13]	*S100 (pg/mL)*	1.782	[0.086,42.126]	*S100 (pg/mL)*	0.810	[0.115,5.683]
*BG (degree 2+3 vs degree 1)*	*α-Synuclein (pg/mL)*	1.001	[0.999,1.002]	*α-Synuclein (pg/mL)*	1.004	[1,1.018]	*α-Synuclein (pg/mL)*	1.000	[0.999,1.002]
*CS (degree 2 vs degree 1)*	*Aβ42/40*	0.001	[0.00001, 0.0017]	*Aβ42/40*			*Aβ42/40*		
*CS (degree 2 vs degree 1)*	*p-Tau (pg/mL)*	0.960	[0.878,1.055]	*p-Tau (pg/mL)*	1.064	[0.905,1.301]	*p-Tau (pg/mL)*	0.824	[0.669,1.003]
*CS (degree 2 vs degree 1)*	*t-Tau (pg/mL)*	0.995	[0.984,1.005]	*t-Tau (pg/mL)*	1.006	[0.986,1.028]	*t-Tau (pg/mL)*	0.984	[0.964,1.004]
*CS (degree 2 vs degree 1)*	*GFAP (pg/mL)*	0.935	[0.806,1.085]	*GFAP (pg/mL)*	1.025	[0.673,1.558]	*GFAP (pg/mL)*	0.921	[0.78,1.095]
*CS (degree 2 vs degree 1)*	*YKL40 (pg/mL)*	0.997	[0.988,1.006]	*YKL40 (pg/mL)*	1.010	[0.99,1.033]	*YKL40 (pg/mL)*	0.992	[0.981,1.003]
*CS (degree 2 vs degree 1)*	*TREM2 (pg/mL)*	0.899	[0.755,1.067]	*TREM2 (pg/mL)*	0.883	[0.636,1.208]	*TREM2 (pg/mL)*	0.911	[0.724,1.143]
*CS (degree 2 vs degree 1)*	*IL6 (pg/mL)*	1.016	[0.866,1.21]	*IL6 (pg/mL)*	1.098	[0.722,1.759]	*IL6 (pg/mL)*	0.991	[0.825,1.212]
*CS (degree 2 vs degree 1)*	*NFL (pg/mL)*	0.992	[0.979,1.002]	*NFL (pg/mL)*	0.992	[0.962,1.024]	*NFL (pg/mL)*	0.992	[0.974,1.002]
*CS (degree 2 vs degree 1)*	*Neurogranin (pg/mL)*	1.001	[0.998,1.003]	*Neurogranin (pg/mL)*	1.003	[0.998,1.009]	*Neurogranin (pg/mL)*	1.000	[0.997,1.004]
*CS (degree 2 vs degree 1)*	*S100 (pg/mL)*	0.960	[0.189,5.014]	*S100 (pg/mL)*	0.445	[0.018,11.609]	*S100 (pg/mL)*	1.155	[0.146,9.506]
*CS (degree 2 vs degree 1)*	*α-Synuclein (pg/mL)*	1.003	[1,1.007]	*α-Synuclein (pg/mL)*	1.013	[0.998,1.038]	*α-Synuclein (pg/mL)*	1.003	[1,1.007]
*CS (degree 3+4 vs degree 1)*	*Aβ42/40*	~0	[0,0.8373]	*Aβ42/40*			*Aβ42/40*		
*CS (degree 3+4 vs degree 1)*	*p-Tau (pg/mL)*	1.010	[0.946,1.112]	*p-Tau (pg/mL)*	1.152	[0.986,1.487]	*p-Tau (pg/mL)*	0.814	[0.643,1.011]
*CS (degree 3+4 vs degree 1)*	*t-Tau (pg/mL)*	1.000	[0.992,1.011]	*t-Tau (pg/mL)*	1.013	[0.996,1.039]	*t-Tau (pg/mL)*	0.982	[0.959,1.003]
*CS (degree 3+4 vs degree 1)*	*GFAP (pg/mL)*	1.021	[0.876,1.217]	*GFAP (pg/mL)*	1.280	[0.77,2.285]	*GFAP (pg/mL)*	1.009	[0.853,1.226]
*CS (degree 3+4 vs degree 1)*	*YKL40 (pg/mL)*	1.002	[0.993,1.012]	*YKL40 (pg/mL)*	1.010	[0.989,1.036]	*YKL40 (pg/mL)*	0.999	[0.988,1.01]
*CS (degree 3+4 vs degree 1)*	*TREM2 (pg/mL)*	1.017	[0.808,1.279]	*TREM2 (pg/mL)*	1.008	[0.643,1.604]	*TREM2 (pg/mL)*	1.051	[0.78,1.421]
*CS (degree 3+4 vs degree 1)*	*IL6 (pg/mL)*	1.118	[0.885,1.429]	*IL6 (pg/mL)*	1.485	[0.898,3.101]	*IL6 (pg/mL)*	1.079	[0.763,1.484]
*CS (degree 3+4 vs degree 1)*	*NFL (pg/mL)*	0.994	[0.982,1.004]	*NFL (pg/mL)*	1.002	[0.972,1.039]	*NFL (pg/mL)*	0.994	[0.977,1.004]
*CS (degree 3+4 vs degree 1)*	*Neurogranin (pg/mL)*	1.001	[0.999,1.004]	*Neurogranin (pg/mL)*	1.003	[0.999,1.009]	*Neurogranin (pg/mL)*	1.000	[0.996,1.005]
*CS (degree 3+4 vs degree 1)*	*S100 (pg/mL)*	0.403	[0.047,3.219]	*S100 (pg/mL)*	1.286	[0.017,113.076]	*S100 (pg/mL)*	0.322	[0.02,4.771]
*CS (degree 3+4 vs degree 1)*	*α-Synuclein (pg/mL)*	1.004	[1,1.011]	*α-Synuclein (pg/mL)*	1.016	[0.999,1.051]	*α-Synuclein (pg/mL)*	1.003	[0.997,1.01]
*CS (degree 3+4 vs degree 2)*	*Aβ42/40*	0.000	[0,0.01349]	*Aβ42/40*			*Aβ42/40*		
*CS (degree 3+4 vs degree 2)*	*p-Tau (pg/mL)*	1.037	[0.981,1.108]	*p-Tau (pg/mL)*	1.158	[1.032,1.351]	*p-Tau (pg/mL)*	0.954	[0.797,1.135]
*CS (degree 3+4 vs degree 2)*	*t-Tau (pg/mL)*	1.004	[0.997,1.012]	*t-Tau (pg/mL)*	1.020	[1.005,1.041]	*t-Tau (pg/mL)*	0.996	[0.977,1.015]
*CS (degree 3+4 vs degree 2)*	*GFAP (pg/mL)*	1.083	[0.963,1.234]	*GFAP (pg/mL)*	1.355	[0.961,2.05]	*GFAP (pg/mL)*	1.065	[0.934,1.229]
*CS (degree 3+4 vs degree 2)*	*YKL40 (pg/mL)*	1.005	[0.997,1.013]	*YKL40 (pg/mL)*	1.006	[0.991,1.022]	*YKL40 (pg/mL)*	1.006	[0.996,1.016]
*CS (degree 3+4 vs degree 2)*	*TREM2 (pg/mL)*	1.112	[0.939,1.322]	*TREM2 (pg/mL)*	1.231	[0.906,1.719]	*TREM2 (pg/mL)*	1.036	[0.831,1.294]
*CS (degree 3+4 vs degree 2)*	*IL6 (pg/mL)*	1.072	[0.904,1.268]	*IL6 (pg/mL)*	1.371	[0.954,2.11]	*IL6 (pg/mL)*	0.993	[0.783,1.217]
*CS (degree 3+4 vs degree 2)*	*NFL (pg/mL)*	1.009	[0.996,1.023]	*NFL (pg/mL)*	1.008	[0.985,1.032]	*NFL (pg/mL)*	1.012	[0.993,1.031]
*CS (degree 3+4 vs degree 2)*	*Neurogranin (pg/mL)*	1.002	[1,1.003]	*Neurogranin (pg/mL)*	1.004	[1.001,1.008]	*Neurogranin (pg/mL)*	1.002	[0.999,1.005]
*CS (degree 3+4 vs degree 2)*	*S100 (pg/mL)*	0.505	[0.094,2.614]	*S100 (pg/mL)*	0.548	[0.028,8.746]	*S100 (pg/mL)*	0.476	[0.051,4.062]
*CS (degree 3+4 vs degree 2)*	*α-Synuclein (pg/mL)*	1.000	[0.999,1.001]	*α-Synuclein (pg/mL)*	1.001	[1,1.004]	*α-Synuclein (pg/mL)*	0.999	[0.996,1.001]

## Discussion

This study aimed to assess whether pathophysiological processes related to AD, as measured by CSF biomarkers, are associated with higher degree of ePVS in a cohort of cognitively unimpaired individuals enriched for positive AD biomarkers.

We found that ePVS in the CS were associated with higher levels of CSF p-tau, t-tau, and neurogranin in Aβ + participants. CSF p-tau, t-tau and neurogranin are widely accepted biomarkers of tau protein pathophysiology, neurodegeneration, and synaptic dysfunction, respectively. These associations between AD biomarkers and ePVS survived after correcting for relevant demographic and cardiovascular risk factors, as well as CSF Aβ40 levels. CSF Aβ40 concentration values are typically used to normalize CSF Aβ42 levels to render a superior performance (Aβ42/40 ratio) in discriminating patients with AD [21, 22]. Since it has been described that ePVS alter CSF dynamics, it could be hypothesized that ePVS may also alter the concentrations of proteins in the CSF. We found significant associations between higher ePVS levels and higher concentrations of CSF Aβ40, independent of Aβ status, thus supporting this hypothesis. In addition, in models not accounting for CSF Aβ40, higher ePVS levels were associated with higher levels of most of the CSF biomarkers, irrespective of the stratification by Aβ status. This finding suggests that levels of these CSF markers may be altered due to impaired CSF clearance and not necessarily due to the involvement of these pathophysiological mechanisms in the development of ePVS. Furthermore, we stratified the analyses in Aβ + and Aβ- individuals to study the associations between ePVS and CSF biomarkers within and outside the Alzheimer’s *continuum*. Therefore, the fact that p-tau, t-tau, and neurogranin survived the correction for CSF Aβ40 levels in Aβ + individuals supports the association of ePVS with core AD pathophysiological mechanisms.

We observed that the risk of ePVS was strongly associated with age. The effect of age was not specific to a given region but was equally significant in both, CS and BG. In turn, the effect was independent of cardiovascular risk factors. Regarding sex differences in the distribution of ePVS, we observed a significant association specific to the CS region, suggesting that women have a higher risk of ePVS in this region dependent on cardiovascular risk factors, perhaps because of specific sex-related mechanisms that also affect the formation of ePVS. Interestingly, we did not find significant results for the BG region, in line with previous work reporting differences in ePVS formation according to the brain region and sex [7, 23].

We additionally found a significant association between risk of ePVS and WMH and systolic blood pressure in BG, as well as WMH and diastolic blood pressure in CS, which are in line with previous studies [24, 25]. Blood pressure effects were age-dependent and disappeared in both regions when correcting for demographic variables. Previous studies have also described strong associations between ePVS with increasing age and hypertension, but despite that, these analyses revealed that when all those variables were considered jointly, only age remained significant, as in the present study [26, 27]. In addition, we found no significant associations between WMH and ePVS after adjusting for other risk factors, which has been also reported recently [6].

Recent studies have hypothesized that additional risk factors (e.g., genetic risk factors) may contribute to a larger portion of extracellular Aβ clearance, influencing the relationship between ePVS and CSF biomarkers [28]. Because *APOE-ε4* enhances Aβ deposition [29], several studies focused on associations between *APOE* genotypes and ePVS, with controversial results. In studies carried out in older individuals and/or high cardiovascular risk, significant differences were found [30, 31]. Contrary to them, some studies performed in healthy younger individuals were in line with our results and did not find significant associations between *APOE* and ePVS [27, 32]. Notice that even though our sample is enriched for *APOE*-*ε*4 carriers, they are relatively young and have a very low cardiovascular risk. In this context, additional studies become essential and relevant for the near future in this field of research.

Several limitations must be considered for this study. Particularly challenging was the evaluation of MRIs with very small ePVS that can be seen as faint, indistinct high signal structures since those can cause a change from one category to another if considered. This difficulty has been previously described [8] and addressed by maintaining a general impression of the region and trying to match it with the categories provided. Moreover, although several PVS rating methods have been developed, we restricted our study to a single rating scale using only a single MRI protocol and limiting the quantification of PVS to two brain regions. Also, dichotomization of PVS scales could cause a substantial reduction in statistical power, thus increasing the change of false negatives in our results. Another limitation stems from the fact that CSF biomarkers showed a high correlation among them in our sample. In particular, p-tau, t-tau, and neurogranin had pair-wise Pearson’s *r* > 0.90. This high correlation might reflect a tight association between tau pathophysiology, AD-related neurodegeneration, and synaptic dysfunction in our sample or be due to other technical or physiological confounding factors. Nevertheless, p-tau measurements obtained with the same essay have been shown to be able to predict clinical decline and conversion to AD [33] and have been approved for clinical use [34]. Finally, even though the associations between ePVS and p-tau, t-tau, and neurogranin in Aβ + individuals survived the correction for Aβ40 levels, Aβ40 cannot be interpreted as a valid marker of overall cerebral protein clearance in physiological conditions. However, the fact that the biomarkers that survive correction for Aβ40 are also significant without it strongly argues for our main result to be robust against this correction.

A strength of our study is the use of high-resolution T2 MRI scans and a small voxel size (1 mm^3^, isotropic), recommended for PVS rating [8]. An additional substantial strength is our sample-based design, which is a large cohort of cognitively unimpaired individuals after an exhaustive neuropsychological and clinical screening procedure. In addition, results are not likely to be severely confounded by comorbidities of dementia, being the individuals of the study at a low mean cardiovascular risk. The studied cohort has a high prevalence of *APOE*-ε4 carriers, thus bringing a higher statistical power in comparison with studies with a similar number of individuals that are genetically closer to the general population [35]. Our sample also has a high prevalence of positive CSF Aβ, individuals compared with other studies.

In conclusion, our findings showed, for the first time, an association between ePVS in the CS and higher levels of CSF core AD biomarkers p-tau and t-tau, as well as CSF neurogranin, in cognitively unimpaired Aβ + individuals. This result supports the association between ePVS and specific AD pathophysiological mechanisms occurring in the early stages of the Alzheimer’s *continuum*.

## Supplementary Information

**Additional file 1: Figure S1.** Association models between Alzheimer’s Disease CSF biomarkers and enlargement of Perivascular Spaces. 

**Additional file 2:** **Table S1**. Characteristics of the sample stratified by Aβ42/40 status across degrees of Perivascular Spaces. Legend: N, sample size; n, count of individuals for each categorical variable; SD, standard deviation; ePVS, enlarged Perivascular Spaces; BG, Basal Ganglia; CS, Centrum Semiovale; CAIDE, Cardiovascular Risk Factors, Aging and Incidence of Dementia; BMI, Body Mass Index; WMH, White Matter Hyperintensities; GM, Gray Matter volume; TIV, Total Intracranial volume. **Table S2**. Individual associations between enlargement of Perivascular Spaces in Basal Ganglia and Centrum Semiovale regions, and demographic and cardiovascular risk factors. Legend: n, count of individuals for each categorical variable; SD, standard deviation; ePVS, enlarged Perivascular Spaces; BG, Basal Ganglia; CS, Centrum Semiovale; CAIDE, Cardiovascular Risk Factors, Aging and Incidence of Dementia; WMH, White Matter Hyperintensities; GM, Gray Matter volume; TIV, Total Intracranial volume. **Table S3**. Associations between Perivascular Spaces in Basal Ganglia and Centrum Semiovale and CSF biomarkers (logistic and multinomial regressions). Models were adjusted by potential demographic and cardiovascular risk factors. Models were stratified by Aβ42/40 positive status. Legend: n, sample size; SD, standard deviation; ePVS, enlarged Perivascular Spaces; BG, Basal Ganglia; CS, Centrum Semiovale; NTK, NeuroToolKit; CSF, cerebrospinal fluid.

## Data Availability

Due to participant’s privacy, individual level data cannot be made publicly available. Researchers who wish to use data of the ALFA study must obtain approval from the ALFA study Management Team.

## Abbreviations

ALFA: ALzheimer and FAmilies
Aβ: β-Amyloid
Aβ40: Amyloid-β 40
Aβ42: Amyloid-β 42
AD: Alzheimer disease
*APOE*: Apolipoprotein E
BG: Basal ganglia
CS: Centrum semiovale
CSF: Cerebrospinal fluid
ePVS: Enlarged perivascular spaces
GM: Gray matter
GFAP: Glial fibrillary acidic protein
IL-6: Interleukin-6
ISF: Brain interstitial fluid
NfL: Neurofilament light
MRI: Magnetic resonance imaging
PVS: Perivascular spaces
p-tau: Phosphorylated tau
sTREM: Soluble triggering receptor expressed on myeloid cells 2 (TREM2)
t-tau: Total tau
TIV: Total intracranial volume
WMH: White matter hyperintensities
