# Supplementary material for: Perivascular spaces are associated with tau pathophysiology and synaptic dysfunction in early Alzheimer’s continuum
Source: Alzheimers Res Ther. 2021 Aug 5;13:135. doi: 10.1186/s13195-021-00878-5 (PMC8340485; doi:10.1186/s13195-021-00878-5)
Supplement: Supplementary file 1 — Additional file 1: Figure S1. Association models between Alzheimer’s Disease CSF biomarkers and enlargement of Perivascular Spaces. [file 13195_2021_878_MOESM1_ESM.docx]

**Supplemental Tables**

**Table S1. Characteristics of the sample stratified by Aβ42/40 status across degrees of Perivascular Spaces.** *Legend: N. sample size; n. count of individuals for each categorical variable; SD. standard deviation; ePVS. enlarged Perivascular Spaces; BG. Basal Ganglia; CS. Centrum Semiovale; CAIDE. Cardiovascular Risk Factors. Aging and Incidence of Dementia; BMI. Body Mass Index; WMH. White Matter Hyperintensities; GM. Gray Matter volume; TIV. Total Intracranial volume.*

|  |  | **Aβ42/40 positivity (*N=122*)** |  | **Aβ42/40 negativity (*N=200*)** |  |
| --- | --- | --- | --- | --- | --- |
|  |  | **Mean (SD) or n(%)** |  | **Mean (SD) or n(%)** | **p-value** |
| Risk factors | *Sex. n(%)* |  |  |  | 0.1673 |
|  | *Male* | 51 (41.8%) |  | 67 (33.5%) |  |
|  | *Female* | 71 (58.2%) |  | 133 (66.5%) |  |
|  | *Age (years)* | 60.9 (5.1) |  | 60.6 (4.5) | 0.552 |
|  | *APOE-ε4 allele status. n(%)* |  |  |  | 5.76E-08 |
|  | *non-carriers* | 87 (71.3%) |  | 121 (60.5%) |  |
|  | *carriers* | 35 (28.7%) |  | 79 (39.5%) |  |
|  | *Education (years)* | 13.8 (3.5) |  | 13.2 (3.5) | 0.092 |
|  | *Diabetes. n(%)* |  |  |  | 1 |
|  | *No* | 108 (96.4%) |  | 186 (96.9%) |  |
|  | *Yes* | 4 (3.6%) |  | 6 (3.1%) |  |
|  | *Dyslipidemia. n(%)* |  |  |  | 0.621 |
|  | *No* | 56 (50%) |  | 103 (53.6%) |  |
|  | *Yes* | 56 (50%) |  | 89 (46.3%) |  |
|  | *BMI (kg/m2)* | 27.1 (4.4) |  | 26.9 (4.1) | 0.952 |
|  | *Systolic Blood Pressure (mmHg)* | 133.2 (15.4) |  | 133 (16.1) | 0.987 |
|  | *Diastolic Blood Pressure(mmHg)* | 76.3 (9.3) |  | 74.9 (10.2) | 0.223 |
|  | *Cholesterol (mg/dL)* | 202.5 (29.6) |  | 204.7 (31.6) | 0.563 |
|  | *Physical Activity. n(%)* |  |  |  | 0.038 |
|  | *non-active* | 27 (25.9%) |  | 28 (15.2%) |  |
|  | *active* | 77 (74.1%) |  | 156 (84.8%) |  |
|  | *CAIDE score (0-15)* | 5.8 (1.7) |  | 5.7 (1.6) | 0.592 |
| Neuroimaging | *Total GM (mm3)* | 590921 (56042.9) |  | 586717 (45940.7) | 0.408 |
|  | *WMH (ml)* | 4.9 (25.1) |  | 2.4 (8.5) | 0.028 |
|  | *TIV (mm3)* | 1453064 (177947.5) |  | 1430292 (166908.9) | 0.29 |
| ePVS | *BG-ePVS* |  |  |  | 0.149 |
|  | *Degree 1* | 69 (56.6%) |  | 93 (46.5%) |  |
|  | *Degree 2+3* | 53 (43.4%) |  | 107 (53.5%) |  |
|  | *CS-ePVS* |  |  |  | 0.185 |
|  | *Degree 1* | 40 (32.8%) |  | 50 (25%) |  |
|  | *Degree 2* | 47 (38.5%) |  | 100 (50%) |  |
|  | *Degree 3+4* | 35 (28.7%) |  | 50 (25%) |  |
| CSF-Biomarkers | *Aβ42 (pg/mL)* | 911.81 (324.6) |  | 1594.3 (582.2) | < 2.2e-16 |
|  | *Aβ40* | 17.81 (4.8) |  | 1792 (4.9) | 0.856 |
|  | *p-Tau (pg/mL)* | 16.15 (9.7) |  | 17.16 (6.2) | 0.0004 |
|  | *t-Tau (pg/mL)* | 185.1 (85.7) |  | 211.7 (63.3) | 4.89 E-07 |
|  | *GFAP (pg/mL)* | 7.5 (3.03) |  | 7.8 (2.5) | 0.2524 |
|  | *YKL40 (pg/mL)* | 139.3 (56.8) |  | 156.8 (53.4) | < 2.2e-16 |
|  | *TREM2 (pg/mL)* | 7.4 (2.2) |  | 8.4 (2.2) | 3.25E-05 |
|  | *IL6 (pg/mL)* | 4.4 (2.3) |  | 3.9 (1.9) | 0.011 |
|  | *NFL (pg/mL)* | 84.6 (34.9) |  | 82.7 (37.8) | 0.837 |
|  | *Neurogranin (pg/mL)* | 699.1 (337.4) |  | 887.2 (319.9) | 3.83E-10 |
|  | *S100 (pg/mL)* | 1.01 (0.28) |  | 1.1 (0.21) | 0.099 |
|  | *α-Synuclein (pg/mL)* | 204.4 (202.3) |  | 269.4 (312.4) | 3.15E-08 |

**Table S2. Individual associations between enlargement of Perivascular Spaces in Basal Ganglia and Centrum Semiovale regions. and demographic and cardiovascular risk factors.** *Legend: n. count of individuals for each categorical variable; SD. standard deviation; ePVS. enlarged Perivascular Spaces; BG. Basal Ganglia; CS. Centrum Semiovale; CAIDE. Cardiovascular Risk Factors. Aging and Incidence of Dementia; WMH. White Matter Hyperintensities; GM. Gray Matter volume; TIV. Total Intracranial volume.*

|  | **BG-ePVS** |  | **CS-ePVS** | | |
| --- | --- | --- | --- | --- | --- |
|  | **OR (95% CI)** |  | **OR (95% CI)** | **OR (95% CI)** | **OR (95% CI)** |
|  | **Degree 2+3 vs Degree 1** |  | **Degree 2 vs Degree 1** | **Degree 3+4 vs Degree 1** | **Degree 3+4 vs Degree 2** |
| *Sex. n(%)* |  |  |  |  |  |
| *Male* | Ref. |  | Ref. | Ref. | Ref. |
| *Female* | 1.43 [0.90;2.26] |  | 1.03 [0.60;1.77] | 1.36 [0.73;2.56] | 1.32 [0.75;2.34] |
| *Age (years)* | **1.13 [1.08;1.19]** |  | 1.03 [0.98;1.09] | **1.11 [1.04;1.19]** | **1.08 [1.01;1.14]** |
| *APOE-e4 allele carriers. n(%)* |  |  |  |  |  |
| *non-carriers* | Ref. |  | Ref. | Ref. | Ref. |
| *heterozygous* | 0.76 [0.48;1.21] |  | 0.93 [0.53;1.63] | 1.19 [0.63;2.24] | 1.28 [0.73;2.25] |
| *homozygous* | 0.58 [0.25;1.27] |  | 0.69 [0.27;1.78] | 0.91 [0.31;2.57] | 1.32 [0.47;3.51] |
| *APOE-e4 allele status. n(%)* |  |  |  |  |  |
| *non-carriers* | Ref. |  | Ref. | Ref. | Ref. |
| *carriers* | 0.72 [0.47;1.12] |  | 0.88 [0.52;1.49] | 1.13 [0.62;2.06] | 1.29 [0.75;2.21] |
| *Education (years)* | 0.97 [0.91;1.03] |  | 1.04 [0.96;1.13] | 0.97 [0.89;1.06] | 0.94 [0.87;1.01] |
| *Diabetes. n(%)* |  |  |  |  |  |
| *No* | Ref. |  | Ref. | Ref. | Ref. |
| *Yes* | 0.62 [0.15;2.29] |  | 0.24 [0.03;1.18] | 0.59 [0.11;2.59] | 2.50 [0.37;21.9] |
| *Dyslipidemia. n(%)* |  |  |  |  |  |
| *No* | Ref. |  | Ref. | Ref. | Ref. |
| *Yes* | 1.24 [0.79;1.95] |  | 1.28 [0.74;2.24] | 1.37 [0.74;2.55] | 1.07 [0.62;1.85] |
| *BMI (kg/m2)* | **0.93 [0.89;0.99]** |  | 0.94 [0.88;1.00] | 0.93 [0.87;1.00] | 0.98 [0.91;1.05] |
| *Systolic Blood Pressure (mmHg)* | **1.02 [1.01;1.03]** |  | 1.01 [0.99;1.02] | 1.02 [1.00;1.03] | 1.01 [0.99;1.03] |
| *Diastolic Blood Pressure(mmHg)* | 0.99 [0.97;1.02] |  | 1.01 [0.98;1.04] | **1.03 [1.00;1.07]** | 1.02 [0.99;1.05] |
| *Cholesterol (mg/dL)* | 1.00 [0.99;1.01] |  | 1.00 [0.99;1.01] | 1.00 [0.99;1.01] | 1.00 [0.99;1.01] |
| *Physical Activity. n(%)* |  |  |  |  |  |
| *non-active* | Ref. |  | Ref. | Ref. | Ref. |
| *active* | 0.57 [0.31;1.04] |  | 0.87 [0.39;1.84] | 0.58 [0.25;1.29] | 0.66 [0.33;1.32] |
| *CAIDE score (0-15)* | 1.12 [0.98;1.30] |  | 0.98 [0.82;1.17] | 1.19 [0.98;1.44] | 1.23 [1.03;1.45] |
| *Total GM (mm3)* | 1.00 [1.00;1.00] |  | 1.00 [1.00;1.00] | 1.00 [1.00;1.00] | 1.00 [1.00;1.00] |
| *WMH (ml)* | **1.23 [1.07;1.42]** |  | 1.14 [0.97;1.36] | **1.33 [1.06;1.67]** | 0.99 [0.96;1.02] |
| *TIV (mm3)* | 1.00 [1.00;1.00] |  | 1.00 [1.00;1.00] | 1.00 [1.00;1.00] | 1.00 [1.00;1.00] |

**Table S3. Associations between Perivascular Spaces in Basal Ganglia and Centrum Semiovale and CSF biomarkers (logistic and multinomial regressions). Models were adjusted by potential demographic and cardiovascular risk factors. Models were stratified by Aβ42/40 positive status.** *Legend: n, sample size; SD, standard deviation; ePVS, enlarged Perivascular Spaces; BG, Basal Ganglia; CS, Centrum Semiovale; NTK, NeuroToolKit; CSF, cerebrospinal fluid.*

|  | **All Individuals** | | | **Aβ42/40 positivity (N=122)** | | | | **Aβ42/40 negativity (N=200)** | | |
| --- | --- | --- | --- | --- | --- | --- | --- | --- | --- | --- |
| *Outcome* | *Biomarker* | *OR* | *IC* | *Biomarker* | *OR* | *IC* |  | *Biomarker* | *OR* | *IC* |
| *BG (degree 2+3 vs degree 1)* | *Aβ40* | 1.152 | [1.078,1.237] | *Aβ40* | 1.161 | [1.012,1.358] |  | *Aβ40* | 1.151 | [1.066,1.253] |
| *BG (degree 2+3 vs degree 1)* | *Aβ42/40* | 0.519 | [0.271, 0.758] | *Aβ42/40* |  |  |  | *Aβ42/40* |  |  |
| *BG (degree 2+3 vs degree 1)* | *p-Tau (pg/mL)* | 1.079 | [1.024,1.146] | *p-Tau (pg/mL)* | 1.072 | [0.997,1.177] |  | *p-Tau (pg/mL)* | 1.110 | [1.028,1.207] |
| *BG (degree 2+3 vs degree 1)* | *t-Tau (pg/mL)* | 1.009 | [1.004,1.014] | *t-Tau (pg/mL)* | 1.009 | [1.001,1.019] |  | *t-Tau (pg/mL)* | 1.011 | [1.004,1.018] |
| *BG (degree 2+3 vs degree 1)* | *GFAP (pg/mL)* | 0.956 | [0.847,1.078] | *GFAP (pg/mL)* | 1.053 | [0.762,1.444] |  | *GFAP (pg/mL)* | 0.945 | [0.826,1.078] |
| *BG (degree 2+3 vs degree 1)* | *YKL40 (pg/mL)* | 1.015 | [1.007,1.024] | *YKL40 (pg/mL)* | 1.013 | [1,1.029] |  | *YKL40 (pg/mL)* | 1.017 | [1.007,1.028] |
| *BG (degree 2+3 vs degree 1)* | *TREM2 (pg/mL)* | 1.230 | [1.065,1.434] | *TREM2 (pg/mL)* | 1.272 | [0.97,1.72] |  | *TREM2 (pg/mL)* | 1.230 | [1.032,1.486] |
| *BG (degree 2+3 vs degree 1)* | *IL6 (pg/mL)* | 1.079 | [0.923,1.274] | *IL6 (pg/mL)* | 1.178 | [0.858,1.643] |  | *IL6 (pg/mL)* | 1.078 | [0.893,1.329] |
| *BG (degree 2+3 vs degree 1)* | *NFL (pg/mL)* | 1.011 | [0.998,1.025] | *NFL (pg/mL)* | 1.038 | [1.008,1.076] |  | *NFL (pg/mL)* | 1.004 | [0.988,1.02] |
| *BG (degree 2+3 vs degree 1)* | *Neurogranin (pg/mL)* | 1.002 | [1.001,1.004] | *Neurogranin (pg/mL)* | 1.003 | [1.001,1.005] |  | *Neurogranin (pg/mL)* | 1.003 | [1.001,1.004] |
| *BG (degree 2+3 vs degree 1)* | *S100 (pg/mL)* | 2.549 | [0.626,10.873] | *S100 (pg/mL)* | 5.236 | [0.378,97.447] |  | *S100 (pg/mL)* | 2.183 | [0.373,13.341] |
| *BG (degree 2+3 vs degree 1)* | *α-Synuclein (pg/mL)* | 1.002 | [1,1.004] | *α-Synuclein (pg/mL)* | 1.006 | [1.001,1.016] |  | *α-Synuclein (pg/mL)* | 1.001 | [0.999,1.003] |
|  |  |  |  |  |  |  |  |  |  |  |
| *Outcome* | *Biomarker* | *OR* | *IC* | *Biomarker* | *OR* | *IC* |  | *Biomarker* | *OR* | *IC* |
| *CS (degree 2 vs degree 1)* | *Aβ40* | 1.091 | [1.017,1.176] | *Aβ40* | 1.121 | [0.96, 1.345] |  | *Aβ40* | 1.079 | [0.995,1.178] |
| *CS (degree 2 vs degree 1)* | *Aβ42/40* | 0.078 | [0.0014, 0.0830] | *Aβ42/40* |  |  |  | *Aβ42/40* |  |  |
| *CS (degree 2 vs degree 1)* | *p-Tau (pg/mL)* | 1.044 | [0.988,1.112] | *p-Tau (pg/mL)* | 1.095 | [0.992,1.253] |  | *p-Tau (pg/mL)* | 1.029 | [0.951,1.122] |
| *CS (degree 2 vs degree 1)* | *t-Tau (pg/mL)* | 1.004 | [0.999,1.01] | *t-Tau (pg/mL)* | 1.008 | [0.998,1.02] |  | *t-Tau (pg/mL)* | 1.004 | [0.997,1.012] |
| *CS (degree 2 vs degree 1)* | *GFAP (pg/mL)* | 1.005 | [0.879,1.157] | *GFAP (pg/mL)* | 1.150 | [0.799,1.68] |  | *GFAP (pg/mL)* | 0.978 | [0.839,1.154] |
| *CS (degree 2 vs degree 1)* | *YKL40 (pg/mL)* | 1.003 | [0.996,1.011] | *YKL40 (pg/mL)* | 1.013 | [0.998,1.031] |  | *YKL40 (pg/mL)* | 0.999 | [0.991,1.008] |
| *CS (degree 2 vs degree 1)* | *TREM2 (pg/mL)* | 1.011 | [0.874,1.173] | *TREM2 (pg/mL)* | 1.001 | [0.765,1.317] |  | *TREM2 (pg/mL)* | 1.029 | [0.853,1.245] |
| *CS (degree 2 vs degree 1)* | *IL6 (pg/mL)* | 1.015 | [0.869,1.201] | *IL6 (pg/mL)* | 1.037 | [0.696,1.614] |  | *IL6 (pg/mL)* | 0.992 | [0.83,1.205] |
| *CS (degree 2 vs degree 1)* | *NFL (pg/mL)* | 0.995 | [0.985,1.004] | *NFL (pg/mL)* | 1.001 | [0.972,1.031] |  | *NFL (pg/mL)* | 0.994 | [0.98,1.004] |
| *CS (degree 2 vs degree 1)* | *Neurogranin (pg/mL)* | 1.001 | [1,1.003] | *Neurogranin (pg/mL)* | 1.003 | [1,1.006] |  | *Neurogranin (pg/mL)* | 1.001 | [1,1.003] |
| *CS (degree 2 vs degree 1)* | *S100 (pg/mL)* | 1.770 | [0.389,8.517] | *S100 (pg/mL)* | 1.156 | [0.067,24.29] |  | *S100 (pg/mL)* | 1.948 | [0.277,14.758] |
| *CS (degree 2 vs degree 1)* | *α-Synuclein (pg/mL)* | 1.004 | [1.001,1.008] | *α-Synuclein (pg/mL)* | 1.010 | [1,1.022] |  | *α-Synuclein (pg/mL)* | 1.004 | [1,1.008] |
|  |  |  |  |  |  |  |  |  |  |  |
| *Outcome* | *Biomarker* | *OR* | *IC* | *Biomarker* | *OR* | *IC* |  | *Biomarker* | *OR* | *IC* |
| *CS (degree 3+4 vs degree 1)* | *Aβ40* | 1.143 | [1.056,1.248] | *Aβ40* | 1.195 | [0.982,1.514] |  | *Aβ40* | 1.145 | [1.046,1.27] |
| *CS (degree 3+4 vs degree 1)* | *Aβ42/40* | 0.002 | [0.00001, 0.006] | *Aβ42/40* |  |  |  | *Aβ42/40* |  |  |
| *CS (degree 3+4 vs degree 1)* | *p-Tau (pg/mL)* | 1.076 | [1.013,1.157] | *p-Tau (pg/mL)* | 1.150 | [1.02,1.388] |  | *p-Tau (pg/mL)* | 1.072 | [0.984,1.179] |
| *CS (degree 3+4 vs degree 1)* | *t-Tau (pg/mL)* | 1.008 | [1.002,1.015] | *t-Tau (pg/mL)* | 1.013 | [1.001,1.031] |  | *t-Tau (pg/mL)* | 1.009 | [1.001,1.017] |
| *CS (degree 3+4 vs degree 1)* | *GFAP (pg/mL)* | 1.099 | [0.932,1.32] | *GFAP (pg/mL)* | 1.359 | [0.864,2.325] |  | *GFAP (pg/mL)* | 1.074 | [0.897,1.325] |
| *CS (degree 3+4 vs degree 1)* | *YKL40 (pg/mL)* | 1.009 | [1.002,1.018] | *YKL40 (pg/mL)* | 1.016 | [0.999,1.039] |  | *YKL40 (pg/mL)* | 1.008 | [0.999,1.017] |
| *CS (degree 3+4 vs degree 1)* | *TREM2 (pg/mL)* | 1.209 | [1.012,1.461] | *TREM2 (pg/mL)* | 1.219 | [0.871,1.798] |  | *TREM2 (pg/mL)* | 1.259 | [0.998,1.631] |
| *CS (degree 3+4 vs degree 1)* | *IL6 (pg/mL)* | 1.108 | [0.882,1.411] | *IL6 (pg/mL)* | 1.601 | [0.966,3.305] |  | *IL6 (pg/mL)* | 1.032 | [0.733,1.414] |
| *CS (degree 3+4 vs degree 1)* | *NFL (pg/mL)* | 0.999 | [0.989,1.008] | *NFL (pg/mL)* | 1.007 | [0.979,1.04] |  | *NFL (pg/mL)* | 0.997 | [0.986,1.008] |
| *CS (degree 3+4 vs degree 1)* | *Neurogranin (pg/mL)* | 1.002 | [1.001,1.004] | *Neurogranin (pg/mL)* | 1.003 | [1,1.007] |  | *Neurogranin (pg/mL)* | 1.002 | [1.001,1.004] |
| *CS (degree 3+4 vs degree 1)* | *S100 (pg/mL)* | 1.253 | [0.206,8.05] | *S100 (pg/mL)* | 1.381 | [0.025,81.956] |  | *S100 (pg/mL)* | 1.288 | [0.134,14.107] |
| *CS (degree 3+4 vs degree 1)* | *α-Synuclein (pg/mL)* | 1.007 | [1.002,1.013] | *α-Synuclein (pg/mL)* | 1.014 | [1.002,1.038] |  | *α-Synuclein (pg/mL)* | 1.007 | [1.001,1.013] |
|  |  |  |  |  |  |  |  |  |  |  |
| *Outcome* | *Biomarker* | *OR* | *IC* | *Biomarker* | *OR* | *IC* |  | *Biomarker* | *OR* | *IC* |
| *CS (degree 3+4 vs degree 2)* | *Aβ40* | 1.071 | [1.003,1.145] | *Aβ40* | 1.075 | [0.952,1.229] |  | *Aβ40* | 1.085 | [1.001,1.181] |
| *CS (degree 3+4 vs degree 2)* | *Aβ42/40* | 0.002 | [0.0011, 0.0053] | *Aβ42/40* |  |  |  | *Aβ42/40* |  |  |
| *CS (degree 3+4 vs degree 2)* | *p-Tau (pg/mL)* | 1.044 | [1.002,1.093] | *p-Tau (pg/mL)* | 1.086 | [1.017,1.186] |  | *p-Tau (pg/mL)* | 1.051 | [0.969,1.142] |
| *CS (degree 3+4 vs degree 2)* | *t-Tau (pg/mL)* | 1.005 | [1.001,1.01] | *t-Tau (pg/mL)* | 1.009 | [1.002,1.019] |  | *t-Tau (pg/mL)* | 1.006 | [0.999,1.014] |
| *CS (degree 3+4 vs degree 2)* | *GFAP (pg/mL)* | 1.117 | [0.992,1.272] | *GFAP (pg/mL)* | 1.382 | [1.014,2.043] |  | *GFAP (pg/mL)* | 1.091 | [0.953,1.265] |
| *CS (degree 3+4 vs degree 2)* | *YKL40 (pg/mL)* | 1.007 | [1.001,1.014] | *YKL40 (pg/mL)* | 1.008 | [0.997,1.021] |  | *YKL40 (pg/mL)* | 1.009 | [1,1.018] |
| *CS (degree 3+4 vs degree 2)* | *TREM2 (pg/mL)* | 1.168 | [1.009,1.362] | *TREM2 (pg/mL)* | 1.258 | [0.971,1.688] |  | *TREM2 (pg/mL)* | 1.124 | [0.927,1.371] |
| *CS (degree 3+4 vs degree 2)* | *IL6 (pg/mL)* | 1.073 | [0.905,1.268] | *IL6 (pg/mL)* | 1.351 | [0.94,2.07] |  | *IL6 (pg/mL)* | 0.999 | [0.785,1.223] |
| *CS (degree 3+4 vs degree 2)* | *NFL (pg/mL)* | 1.012 | [1,1.026] | *NFL (pg/mL)* | 1.011 | [0.991,1.035] |  | *NFL (pg/mL)* | 1.016 | [0.999,1.035] |
| *CS (degree 3+4 vs degree 2)* | *Neurogranin (pg/mL)* | 1.001 | [1,1.002] | *Neurogranin (pg/mL)* | 1.002 | [1,1.004] |  | *Neurogranin (pg/mL)* | 1.002 | [1,1.003] |
| *CS (degree 3+4 vs degree 2)* | *S100 (pg/mL)* | 1.062 | [0.236,4.746] | *S100 (pg/mL)* | 1.208 | [0.101,14.178] |  | *S100 (pg/mL)* | 1.098 | [0.143,7.973] |
| *CS (degree 3+4 vs degree 2)* | *α-Synuclein (pg/mL)* | 1.000 | [0.999,1.001] | *α-Synuclein (pg/mL)* | 1.001 | [1,1.004] |  | *α-Synuclein (pg/mL)* | 1.000 | [0.997,1.001] |

**Table S4. Associations Perivascular Spaces in Basal Ganglia and Centrum Semiovale and CSF biomarkers (logistic and multinomial regressions). Models were adjusted by potential demographic and cardiovascular risk factors, as well as, levels of Aβ40. Models were stratified by Aβ42/40 positive status.** *Legend: n, sample size; SD, standard deviation; ePVS, enlarged Perivascular Spaces; BG, Basal Ganglia; CS, Centrum Semiovale; NTK, NeuroToolKit; CSF, cerebrospinal fluid.*

|  | **All Individuals** | | | **Aβ42/40 positivity (N=122)** | | | | **Aβ42/40 negativity (N=200)** | | |
| --- | --- | --- | --- | --- | --- | --- | --- | --- | --- | --- |
| *Outcome* | *Biomarker* | *OR* | *IC* | *Biomarker* | *OR* | *IC* |  | *Biomarker* | *OR* | *IC* |
| *BG (degree 2+3 vs degree 1)* | *Aβ42/40* | 0.059 | [0.025, 0.626] | *Aβ42/40* |  |  |  | *Aβ42/40* |  |  |
| *BG (degree 2+3 vs degree 1)* | *p-Tau (pg/mL)* | 0.980 | [0.925,1.055] | *p-Tau (pg/mL)* | 1.022 | [0.937,1.161] |  | *p-Tau (pg/mL)* | 0.887 | [0.74,1.061] |
| *BG (degree 2+3 vs degree 1)* | *t-Tau (pg/mL)* | 0.999 | [0.991,1.008] | *t-Tau (pg/mL)* | 1.004 | [0.992,1.022] |  | *t-Tau (pg/mL)* | 0.992 | [0.974,1.011] |
| *BG (degree 2+3 vs degree 1)* | *GFAP (pg/mL)* | 0.883 | [0.762,0.998] | *GFAP (pg/mL)* | 0.873 | [0.589,1.254] |  | *GFAP (pg/mL)* | 0.895 | [0.758,1.021] |
| *BG (degree 2+3 vs degree 1)* | *YKL40 (pg/mL)* | 1.008 | [0.999,1.018] | *YKL40 (pg/mL)* | 1.006 | [0.987,1.026] |  | *YKL40 (pg/mL)* | 1.010 | [0.999,1.023] |
| *BG (degree 2+3 vs degree 1)* | *TREM2 (pg/mL)* | 1.082 | [0.917,1.285] | *TREM2 (pg/mL)* | 1.127 | [0.814,1.583] |  | *TREM2 (pg/mL)* | 1.072 | [0.873,1.325] |
| *BG (degree 2+3 vs degree 1)* | *IL6 (pg/mL)* | 1.108 | [0.943,1.324] | *IL6 (pg/mL)* | 1.192 | [0.854,1.708] |  | *IL6 (pg/mL)* | 1.119 | [0.918,1.413] |
| *BG (degree 2+3 vs degree 1)* | *NFL (pg/mL)* | 1.000 | [0.986,1.014] | *NFL (pg/mL)* | 1.031 | [0.998,1.07] |  | *NFL (pg/mL)* | 0.991 | [0.974,1.009] |
| *BG (degree 2+3 vs degree 1)* | *Neurogranin (pg/mL)* | 1.001 | [0.999,1.004] | *Neurogranin (pg/mL)* | 1.002 | [0.999,1.007] |  | *Neurogranin (pg/mL)* | 1.001 | [0.998,1.005] |
| *BG (degree 2+3 vs degree 1)* | *S100 (pg/mL)* | 0.850 | [0.177,4.13] | *S100 (pg/mL)* | 1.782 | [0.086,42.126] |  | *S100 (pg/mL)* | 0.810 | [0.115,5.683] |
| *BG (degree 2+3 vs degree 1)* | *α-Synuclein (pg/mL)* | 1.001 | [0.999,1.002] | *α-Synuclein (pg/mL)* | 1.004 | [1,1.018] |  | *α-Synuclein (pg/mL)* | 1.000 | [0.999,1.002] |
|  |  |  |  |  |  |  |  |  |  |  |
| *Outcome* | *Biomarker* | *OR* | *IC* | *Biomarker* | *OR* | *IC* |  | *Biomarker* | *OR* | *IC* |
| *CS (degree 2 vs degree 1)* | *Aβ42/40* | 0.001 | [0.00001, 0.0017] | *Aβ42/40* |  |  |  | *Aβ42/40* |  |  |
| *CS (degree 2 vs degree 1)* | *p-Tau (pg/mL)* | 0.960 | [0.878,1.055] | *p-Tau (pg/mL)* | 1.064 | [0.905,1.301] |  | *p-Tau (pg/mL)* | 0.824 | [0.669,1.003] |
| *CS (degree 2 vs degree 1)* | *t-Tau (pg/mL)* | 0.995 | [0.984,1.005] | *t-Tau (pg/mL)* | 1.006 | [0.986,1.028] |  | *t-Tau (pg/mL)* | 0.984 | [0.964,1.004] |
| *CS (degree 2 vs degree 1)* | *GFAP (pg/mL)* | 0.935 | [0.806,1.085] | *GFAP (pg/mL)* | 1.025 | [0.673,1.558] |  | *GFAP (pg/mL)* | 0.921 | [0.78,1.095] |
| *CS (degree 2 vs degree 1)* | *YKL40 (pg/mL)* | 0.997 | [0.988,1.006] | *YKL40 (pg/mL)* | 1.010 | [0.99,1.033] |  | *YKL40 (pg/mL)* | 0.992 | [0.981,1.003] |
| *CS (degree 2 vs degree 1)* | *TREM2 (pg/mL)* | 0.899 | [0.755,1.067] | *TREM2 (pg/mL)* | 0.883 | [0.636,1.208] |  | *TREM2 (pg/mL)* | 0.911 | [0.724,1.143] |
| *CS (degree 2 vs degree 1)* | *IL6 (pg/mL)* | 1.016 | [0.866,1.21] | *IL6 (pg/mL)* | 1.098 | [0.722,1.759] |  | *IL6 (pg/mL)* | 0.991 | [0.825,1.212] |
| *CS (degree 2 vs degree 1)* | *NFL (pg/mL)* | 0.992 | [0.979,1.002] | *NFL (pg/mL)* | 0.992 | [0.962,1.024] |  | *NFL (pg/mL)* | 0.992 | [0.974,1.002] |
| *CS (degree 2 vs degree 1)* | *Neurogranin (pg/mL)* | 1.001 | [0.998,1.003] | *Neurogranin (pg/mL)* | 1.003 | [0.998,1.009] |  | *Neurogranin (pg/mL)* | 1.000 | [0.997,1.004] |
| *CS (degree 2 vs degree 1)* | *S100 (pg/mL)* | 0.960 | [0.189,5.014] | *S100 (pg/mL)* | 0.445 | [0.018,11.609] |  | *S100 (pg/mL)* | 1.155 | [0.146,9.506] |
| *CS (degree 2 vs degree 1)* | *α-Synuclein (pg/mL)* | 1.003 | [1,1.007] | *α-Synuclein (pg/mL)* | 1.013 | [0.998,1.038] |  | *α-Synuclein (pg/mL)* | 1.003 | [1,1.007] |
|  |  |  |  |  |  |  |  |  |  |  |
| *Outcome* | *Biomarker* | *OR* | *IC* | *Biomarker* | *OR* | *IC* |  | *Biomarker* | *OR* | *IC* |
| *CS (degree 3+4 vs degree 1)* | *Aβ42/40* | ~0 | [0,0.8373] | *Aβ42/40* |  |  |  | *Aβ42/40* |  |  |
| *CS (degree 3+4 vs degree 1)* | *p-Tau (pg/mL)* | 1.010 | [0.946,1.112] | *p-Tau (pg/mL)* | 1.152 | [0.986,1.487] |  | *p-Tau (pg/mL)* | 0.814 | [0.643,1.011] |
| *CS (degree 3+4 vs degree 1)* | *t-Tau (pg/mL)* | 1.000 | [0.992,1.011] | *t-Tau (pg/mL)* | 1.013 | [0.996,1.039] |  | *t-Tau (pg/mL)* | 0.982 | [0.959,1.003] |
| *CS (degree 3+4 vs degree 1)* | *GFAP (pg/mL)* | 1.021 | [0.876,1.217] | *GFAP (pg/mL)* | 1.280 | [0.77,2.285] |  | *GFAP (pg/mL)* | 1.009 | [0.853,1.226] |
| *CS (degree 3+4 vs degree 1)* | *YKL40 (pg/mL)* | 1.002 | [0.993,1.012] | *YKL40 (pg/mL)* | 1.010 | [0.989,1.036] |  | *YKL40 (pg/mL)* | 0.999 | [0.988,1.01] |
| *CS (degree 3+4 vs degree 1)* | *TREM2 (pg/mL)* | 1.017 | [0.808,1.279] | *TREM2 (pg/mL)* | 1.008 | [0.643,1.604] |  | *TREM2 (pg/mL)* | 1.051 | [0.78,1.421] |
| *CS (degree 3+4 vs degree 1)* | *IL6 (pg/mL)* | 1.118 | [0.885,1.429] | *IL6 (pg/mL)* | 1.485 | [0.898,3.101] |  | *IL6 (pg/mL)* | 1.079 | [0.763,1.484] |
| *CS (degree 3+4 vs degree 1)* | *NFL (pg/mL)* | 0.994 | [0.982,1.004] | *NFL (pg/mL)* | 1.002 | [0.972,1.039] |  | *NFL (pg/mL)* | 0.994 | [0.977,1.004] |
| *CS (degree 3+4 vs degree 1)* | *Neurogranin (pg/mL)* | 1.001 | [0.999,1.004] | *Neurogranin (pg/mL)* | 1.003 | [0.999,1.009] |  | *Neurogranin (pg/mL)* | 1.000 | [0.996,1.005] |
| *CS (degree 3+4 vs degree 1)* | *S100 (pg/mL)* | 0.403 | [0.047,3.219] | *S100 (pg/mL)* | 1.286 | [0.017,113.076] |  | *S100 (pg/mL)* | 0.322 | [0.02,4.771] |
| *CS (degree 3+4 vs degree 1)* | *α-Synuclein (pg/mL)* | 1.004 | [1,1.011] | *α-Synuclein (pg/mL)* | 1.016 | [0.999,1.051] |  | *α-Synuclein (pg/mL)* | 1.003 | [0.997,1.01] |
|  |  |  |  |  |  |  |  |  |  |  |
| *Outcome* | *Biomarker* | *OR* | *IC* | *Biomarker* | *OR* | *IC* |  | *Biomarker* | *OR* | *IC* |
| *CS (degree 3+4 vs degree 2)* | *Aβ42/40* | 0.000 | [0,0.01349] | *Aβ42/40* |  |  |  | *Aβ42/40* |  |  |
| *CS (degree 3+4 vs degree 2)* | *p-Tau (pg/mL)* | 1.037 | [0.981,1.108] | *p-Tau (pg/mL)* | 1.158 | [1.032,1.351] |  | *p-Tau (pg/mL)* | 0.954 | [0.797,1.135] |
| *CS (degree 3+4 vs degree 2)* | *t-Tau (pg/mL)* | 1.004 | [0.997,1.012] | *t-Tau (pg/mL)* | 1.020 | [1.005,1.041] |  | *t-Tau (pg/mL)* | 0.996 | [0.977,1.015] |
| *CS (degree 3+4 vs degree 2)* | *GFAP (pg/mL)* | 1.083 | [0.963,1.234] | *GFAP (pg/mL)* | 1.355 | [0.961,2.05] |  | *GFAP (pg/mL)* | 1.065 | [0.934,1.229] |
| *CS (degree 3+4 vs degree 2)* | *YKL40 (pg/mL)* | 1.005 | [0.997,1.013] | *YKL40 (pg/mL)* | 1.006 | [0.991,1.022] |  | *YKL40 (pg/mL)* | 1.006 | [0.996,1.016] |
| *CS (degree 3+4 vs degree 2)* | *TREM2 (pg/mL)* | 1.112 | [0.939,1.322] | *TREM2 (pg/mL)* | 1.231 | [0.906,1.719] |  | *TREM2 (pg/mL)* | 1.036 | [0.831,1.294] |
| *CS (degree 3+4 vs degree 2)* | *IL6 (pg/mL)* | 1.072 | [0.904,1.268] | *IL6 (pg/mL)* | 1.371 | [0.954,2.11] |  | *IL6 (pg/mL)* | 0.993 | [0.783,1.217] |
| *CS (degree 3+4 vs degree 2)* | *NFL (pg/mL)* | 1.009 | [0.996,1.023] | *NFL (pg/mL)* | 1.008 | [0.985,1.032] |  | *NFL (pg/mL)* | 1.012 | [0.993,1.031] |
| *CS (degree 3+4 vs degree 2)* | *Neurogranin (pg/mL)* | 1.002 | [1,1.003] | *Neurogranin (pg/mL)* | 1.004 | [1.001,1.008] |  | *Neurogranin (pg/mL)* | 1.002 | [0.999,1.005] |
| *CS (degree 3+4 vs degree 2)* | *S100 (pg/mL)* | 0.505 | [0.094,2.614] | *S100 (pg/mL)* | 0.548 | [0.028,8.746] |  | *S100 (pg/mL)* | 0.476 | [0.051,4.062] |
| *CS (degree 3+4 vs degree 2)* | *α-Synuclein (pg/mL)* | 1.000 | [0.999,1.001] | *α-Synuclein (pg/mL)* | 1.001 | [1,1.004] |  | *α-Synuclein (pg/mL)* | 0.999 | [0.996,1.001] |
